# Supplementary material for: Stochastic Constitutive Model of Isotropic Thin Fiber Networks Based on Stochastic Volume Elements
Source: Materials (Basel). 2019 Feb 11;12(3):538. doi: 10.3390/ma12030538 (PMC6384688; doi:10.3390/ma12030538)
Supplement: Supplementary file 1 [file materials-12-00538-s001.zip › Suppli/Borodulina_et_al_effect_of_fiber_and_bond_variations_AK.pdf]

Effect of fiber and bond strength variations on the tensile stiffness and strength of fiber networks

S. Borodulina*, H. R. Motamedian, A. Kulachenko

Department of Solid Mechanics, Royal Institute of Technology (KTH), SE-100 44 Stockholm, Sweden

Abstract

As fiber and bond characterization tools become more sophisticated, the information from the fiber scale becomes richer. This information is used for benchmarking of different types of fibers by the paper and packaging industries. In this work, we have addressed a question about the effect of variability in the fiber and fiber bond properties on the average stiffness and strength of fiber networks. We used a fiber-scale numerical model and reconstruction algorithm to address this question. The approach was verified using the experimental sheets having fiber data acquired by a fiber morphology analyzer and corrected by microtomographic analysis of fibers in these sheets. We concluded, among other things, that it is sufficient to account for the average bond strength value with an acceptable number of samples to describe dry network strength, as long as the bond strength distribution remains symmetric. We also found that using the length-weighted average for fiber shape factor and fiber length data neglects the important contribution from the distribution in these properties on the mechanical properties of the sheets.

Keywords: fibers, bonds, stress-strain curve, paper strength, network simulation

1. Introduction

Man-made materials with a fiber network structure have a clear advantage over other materials in favor of a controlled anisotropy, which makes it possible to enhance the mechanical properties in the relevant direction. For example, in paper and packaging products, the in-plane fiber orientation ensures a large in-plane stiffness to mass ratio.

The in-plane strength of the dry paper material has remained a central theme in papermaking research for many years. It is generally accepted that the tensile strength of paper can be described by the mean properties of its constituents, which are the mechanical properties of fibers and fiber bonds, the relative bonded area (RBA) between the fibers, and fiber orientation (Alava and Niskanen, 2006). The tensile index (*T,* tensile strength per density) for a paper sheet with uniform random fiber orientation is usually characterized by the Page equation (Page, 1969):

* Corresponding author. Tel. +46 8790 8644; fax: + 46 9790 9189. *Email address*: svebor@kth.se (S. Borodulina)

$$\frac{1}{T} = \underbrace{\frac{9}{8Z}}_{\text{Contribution of fibers}} + \underbrace{\frac{1}{B}}_{\text{Contribution of bonding}}, \tag{1}$$

where T, $Z^{\dagger}$ and B have dimensions of N·m·kg^{-1}. In this equation, the contribution of fibers' length (l_f) and width (w_f), breaking stress of bonds (τ_b), as well as RBA in bonded fibers, is merged into a single variable B ($1/B = 3w_f / l_f \tau_b RBA$).

Indeed, in the characterization of paper constituents, both the bond and fiber characteristics are traditionally used in terms of mean values rather than distributions in such empirical relations. In fact, the fiber morphological properties are non-uniform with the fiber lengths and cross-sectional properties being subject to natural variation. The distribution of fiber properties can be characterized using modern high-throughput tools in a wet state (Hirn and Bauer, 2006) and X-ray tomography in a dry state (Marulier et al., 2015; Borodulina et al., 2016). Similarly, the bond strength exhibits a large variation depending on the geometry of the bond and the bonded area. Finally, the structure of the paper network itself is clearly heterogeneous. Here, we will discuss the effect of such a variation. We limit ourselves to the effects of the variability of certain bond and fiber characteristics on the in-plane stiffness, strength, and strain to failure of the fiber network.

The nature of fiber bonding creates notorious difficulties in sample preparation and testing methods. Up to the present, a number of experimental methods and techniques have been proposed for measuring bond strength between two isolated fibers. A large scatter in data is reported depending on the measuring procedure (Skowronski, 1991; Eriksson et al., 2006; Rohm et al., 2014), to mention a few.

Recently, Marais et al. (2014) used polyamines dry-strength additives to alter the surface of the fibers in order to understand the mechanisms behind the strength enhancement. They presented experimental results on the strength measurement of interfiber joints with and without fiber modification, with the distribution of interfiber joint strengths following a heavy-tailed Weibull- or exponential distribution. It was unclear whether the difference in distribution could contribute to the considerable effects introduced into the mechanical properties on the sheet level (Marais and Wågberg, 2012).

In the same fashion, automatic measurements of fiber morphologies may give differences in fiber length of up to 20% (Carvalho et al., 1997) in the data for the same pulp, depending on the technique and the definition of the average values (viz. numerical mean, length-weighted or weight-weighted) adopted. As a result, length-weighted data are more commonly used, since it does not significantly depend on the fraction of fines‡, and correlates better with paper properties (Paavilainen, 1990).

Here, we study numerically the effect of various bond strength distributions in relation to mechanical properties of paper. We resort to utilizing the fiber network model presented in our earlier studies (Kulachenko and Uesaka, 2012; Borodulina

---

† Zero span tensile index that characterizes strength of fibers (Bronkhorst and Bennet, 2002)

‡ Small fractions of pulp with lengths shorter than 0.2 mm

et al., 2012). In addition to bond properties, we study the effect of variability in fiber morphologies, which can readily be captured, but is rarely used in terms of distribution. For this work, we developed the procedure of reconstructing the three-dimensional (3D) network to ensure that the structural characteristics of the networks resemble the experimental sheets used in the verification of the model.

2. Methods

We model paper as a 3D network of interconnected fibers. There have been a number of studies approaching paper material on this scale using two-dimensional fiber networks (Räisänen et al., 1996; Niskanen et al., 1999; Hägglund and Isaksson, 2008). A full 3D network model is required for a more realistic representation of paper structure, accurate estimation of the bonds, and capturing the energy stored in out-of-plane deformation of the fibers and fiber bonds. Few steps have been taken in modeling 3D fiber networks (Wang and Shaler, 1998; Nilsen and Zabihian, 1998; Heyden, 2000; Kulachenko and Uesaka, 2012; Wilbrink et al., 2013). For this particular study, the principal advantage of 3D models is the ability to assess the contribution of real fiber data to the fiber connectivity inside the network. In addition, we endeavor to provide a thoughtful description of the mechanical and structural aspects of the 3D fiber networks, which will enable us to verify it against the physical tests performed on the handsheets.

2.1. Network reconstruction

2.1.1. Fibers

In order to simulate the paper material, we need to define a network of fibers with appropriate fiber geometries, orientations, and material properties. We consider a network of bonded dry fibers.

We form the network of fibers numerically by a deposition technique (Kulachenko and Uesaka, 2012). For this purpose, we use fiber geometry data extracted by automatic fiber morphology analysis (FMA) characterization tools (Hirn and Bauer, 2006) in the wet state. As the fiber cross-section changes significantly upon pressing and drying, we refine the measured data with the help of X-ray tomography scans of the dry sheets.

For all the measurements, we used the same unbleached commercial softwood kraft fiber pulp with removed fiber fractions that are smaller than 0.2 mm. These fractions were removed by mesh screening. Kraft fibers are usually chemically separated from wood; this process dissolves the phenolic resin (lignin) in the cell walls of fibers, making them flexible, resulting in overall good bonding properties. In all the computations, the fiber density was assumed to be 1430 kg/m^3.

In our model, each fiber is represented as a series of 3-node quadratic, geometrically non-linear Timoshenko/Reissner beam elements (Ibrahimbegovic, 1995) with either solid or hollow rectangular cross-sections since fibers may have a non-circular or collapsed cross-section. After a mesh convergence study, the resulting element size was set to be 40 μm. The section properties were computed at two quadrature stations along the beam by the Gaussian integration over the

discretized beam cross-section (Fig. 1). The constitutive relations were evaluated at every integration point. Any coupling between torsional and axial deformation due to warping effects was neglected.

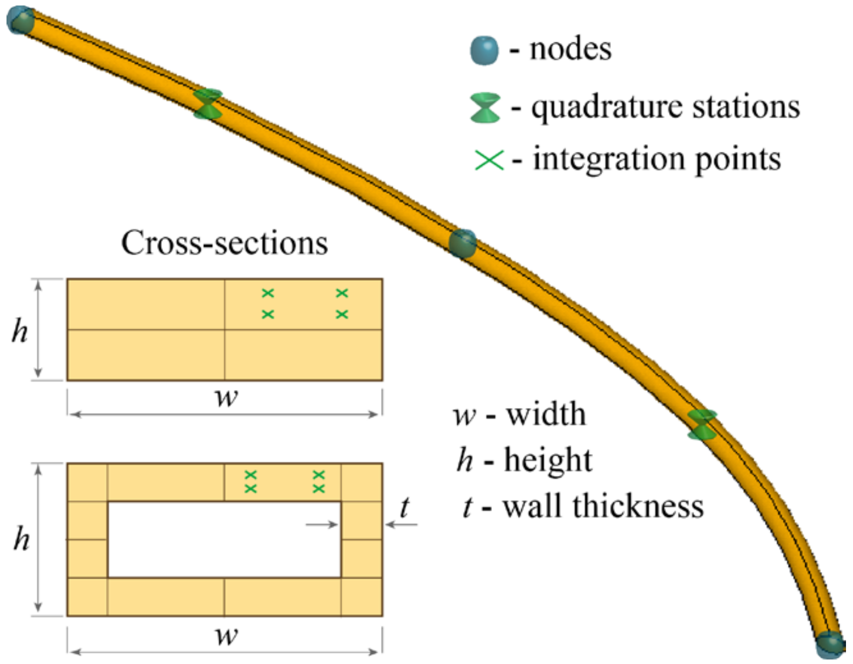

Fig. 1 Beam element topology and supported fiber cross-sections with depicted quadrature stations and integration points.

The finite element equations were solved by the implicit time integration scheme in a quasi-static regime. Prior to simulation and at the end of every converged sub step, we scanned the network for fibers that have one single or no connection at all to any other fiber. Additionally, isolated fiber islands (and all the elements forming these), which were not connected to the constrained fiber network, were eliminated from the system in order to alleviate ill-conditioned global stiffness matrix and convergence problems associated with it.

Fig. 3 shows the extracted fiber geometry data from FMA, in terms of distribution of fiber width and fiber shape factor (i.e. the ratio between fiber end points AOB and fiber length AOB, schematically shown in Fig. 2). These geometrical data for fibers have been used as input parameters for network generation. Considering that the original fiber width from FMA is from the wet state, it is corrected according to µCT measurements of width and height for each cross-section. The fiber shape factor and fiber length data do not need to be corrected since the fiber length does not change considerably during paper drying.

The cross-section characteristics of the fibers are adjusted by a cross-sectional shrinkage factor for each individual fiber and accompanied by appropriate width-to-height ratios.

$$\text{Fiber shape factor } c = \frac{\|AO'B\|}{\|AOB\|}$$

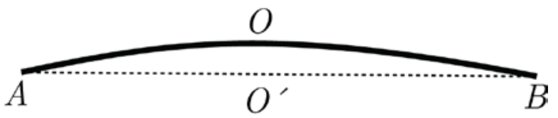

Fig. 2. Definition of fiber shape factor.

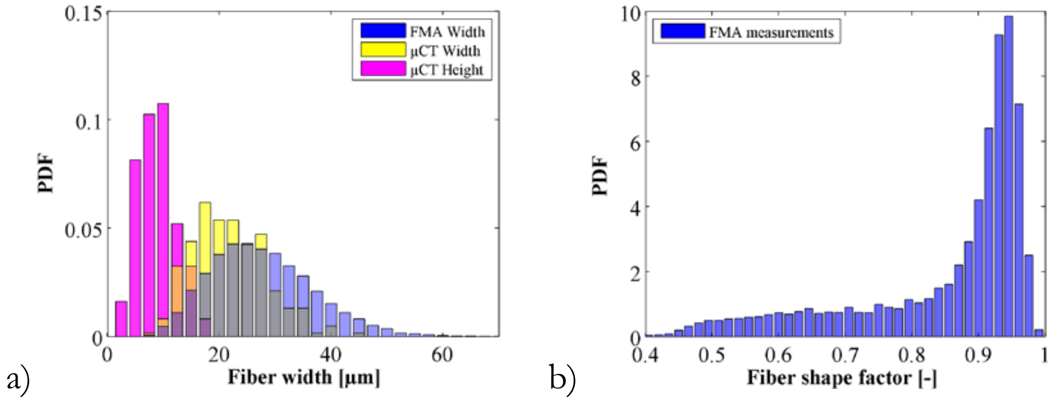

Fig. 3. Fiber geometrical characteristics: a) fiber width extracted with fiber morphology analyzer (FMA) tools, and fiber width and height from micro-computed tomography (μCT), b) fiber shape factor from FMA. FMA measurements were performed on wet pulp; μCT was done on dry sheets. The heights of the bars have been normalized to give a unit area in total.

In order to correct the cross-sectional data, we processed the scanned images of the sheets made of the considered kraft pulp using the micro-computed tomography (μCT) methods described elsewhere (Wernersson et al., 2014; Borodulina et al., 2016). These methods enable the extraction of images of fiber cross-sections in the dry state. From image analysis methods, we extract orientation, width, height, wall thickness and total area for each of the cross-sections. We then use these data to fit a rectangle. The range of possible cross-sections and fitted rectangles is demonstrated in Fig. 4. This rectangle is in terms of width-to-height ratio (WH ratio) and fiber wall thickness, schematically illustrated in Fig. 4g. Typically, a large fraction of the fibers has nearly closed or collapsed cross-sections.

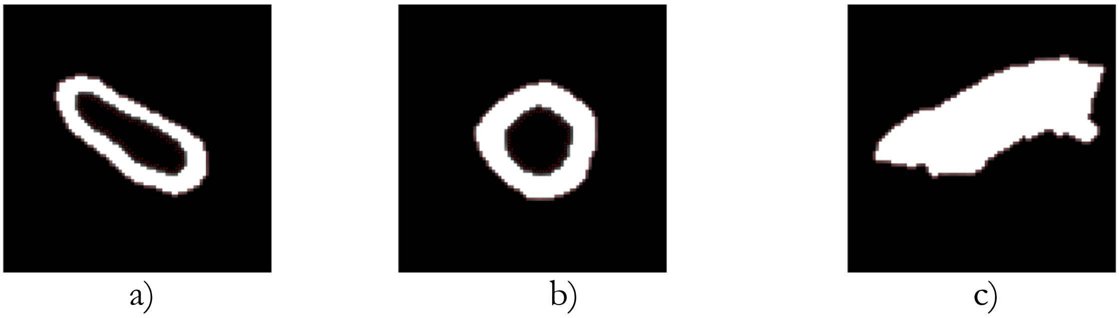

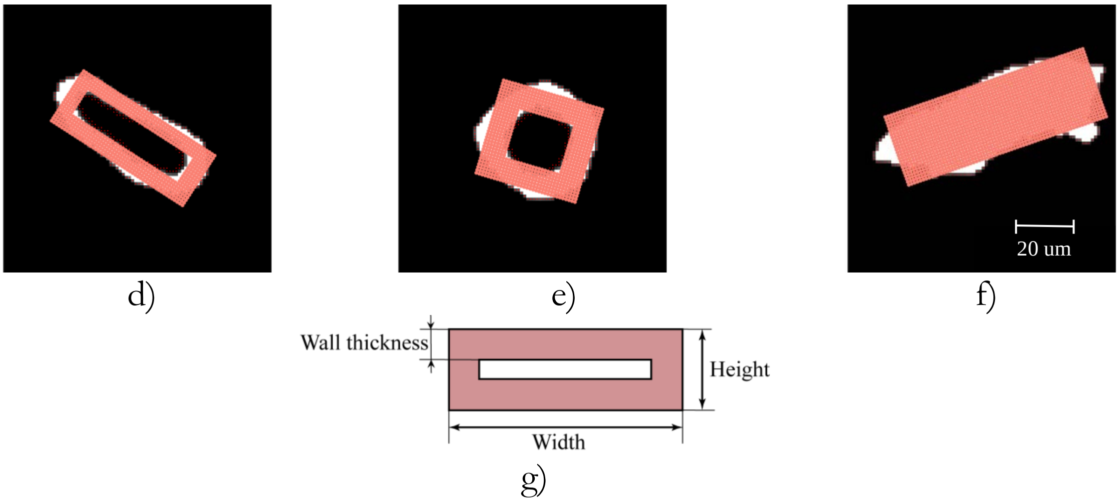

Fig. 4. Images of the actual fiber cross-section in paper: a,b) with and c) without a lumen, computed from X-ray tomograms. Geometrical representation of the same cross-sections defined by a rectangle with hollow d), e) and solid f) cross-sections. g) Model definition of the fiber geometrical characteristics for the cross-section with WH ratio of 2.9 (the computed average proportions are preserved).

For the considered pulp, the mean WH ratio was 2.9 with a standard deviation of 1.78. Having cross-section information in this format gives us appropriate reference values for the numerical model. The detailed fiber data are summarized in Table 1.

Table 1. Fiber geometrical data used in the network simulation, based on the direct measurement on wet pulp (FMA), on dry sheets (μCT) and numerical parameters in terms of length-weighted mean and standard deviation (SD) values.

	**Mean**	**SD**	**Source of data**
Fiber length, mm	2.34	0.90	FMA
Fiber width, μm	23.83	7.09	FMA corrected by μCT
Fiber wall thickness, μm	3.96	1.90	FMA corrected by μCT
WH ratio, [-]	2.9	1.72	μCT
Fiber shape factor	0.945	0.015	FMA
Maximum interface angle[§], °	5	-	Numerical
Radius swelling factor[**], [-]	0.78	0.68	μCT
Wall thickness swelling factor[*], [-]	0. 528	0.31	μCT

The constitutive response of the fibers was described by a bilinear plasticity model with the material parameters as listed in Table 2:

Table 2. Fiber material parameters used in the network simulation.

**Elastic modulus, GPa**	**Tangent modulus, GPa**	**Yield stress, MPa**
30	10	150

[§] Defined in Fig. 7.

[**] The ratio between dry and wet measured radius and wall thickness, respectively.

2.1.2. Contact formulation

A fiber-to-fiber bond is treated as a point contact between three-dimensional beams using a beam-to-beam contact formulation. In this formulation, bonds are treated as inseparable (adhesive) contact points. To detect the contact between the fibers, we used minimum distance algorithm (closest projection method), which iteratively solves a system of non-linear equations (Kulachenko and Uesaka, 2012). In this algorithm, the cross-sections of the fibers are treated as circular and rigid, with the diameter equal to the mean of the values of fiber width and height. This simplification makes the contact search algorithm very effective. The algorithms, which are capable of treating rectangular fibers are available in the literature, *e.g.* (Litewka and Wriggers, 2002). However, in the case of bonded fibers described by the point-wise contact, which captures the exact fiber cross-section during the contact search, these algorithms do not bring any considerable changes, but may affect the number of detected contacts.

Although the same contact detection algorithm is used during generation and computation of the network, the discretization is changed after cutting the network to the target size. As a result of the changed discretization, initial interpenetration is detected in a number of contact interfaces even prior to loading phase. The initial interpenetration is recorded and is used as a permanent offset for the contact pairs. In this way, we ensured that the detected contacts with initial interpenetration appeared to be in the perfect contact and did not cause convergence problems.

To force the inseparable contact constraint, one normal and two orthogonal tangential as well as rotational gaps are defined as follows:

$$
\begin{aligned}
u_{\mathrm{n}} &= d-(r_1+r_2) \\
u_{\mathrm{t}}^{1} &= \frac{1}{2} l_1(\xi_1^{\mathrm{c}}-\xi_1^{\mathrm{o}}) \\
\theta_{\mathrm{n}} &= \theta_{\mathrm{c}}-\theta_{\mathrm{o}} = \mathbf{t}_1^{\mathrm{c}}\cdot\mathbf{t}_2^{\mathrm{c}}-\mathbf{t}_1^{\mathrm{o}}\cdot\mathbf{t}_2^{\mathrm{o}}
\end{aligned}
\qquad (2)
$$

where u_{n} is the normal gap, r_1 and r_2 are the radii of beams in contact, d is the minimum distance between the centerlines of the beams determined by the contact search algorithm; u_{t}^{1} is the tangential gap along the master beam, l_1 is the length of the master beam, ξ_1^{c} and ξ_1^{o} are current and initial dimensionless positions of the contact point along beam i respectively ($\xi_i \in [-1,1]$); θ_{n} is the in-plane rotational gap, θ_{c} and θ_{o} are current and initial angles between the tangents to the beams at the contact point, and $\mathbf{t}_i^{\mathrm{c}}$ and $\mathbf{t}_i^{\mathrm{o}}$ are current and initial unit tangent vectors to beam i at the contact point. These parameters are shown in Fig. 5.

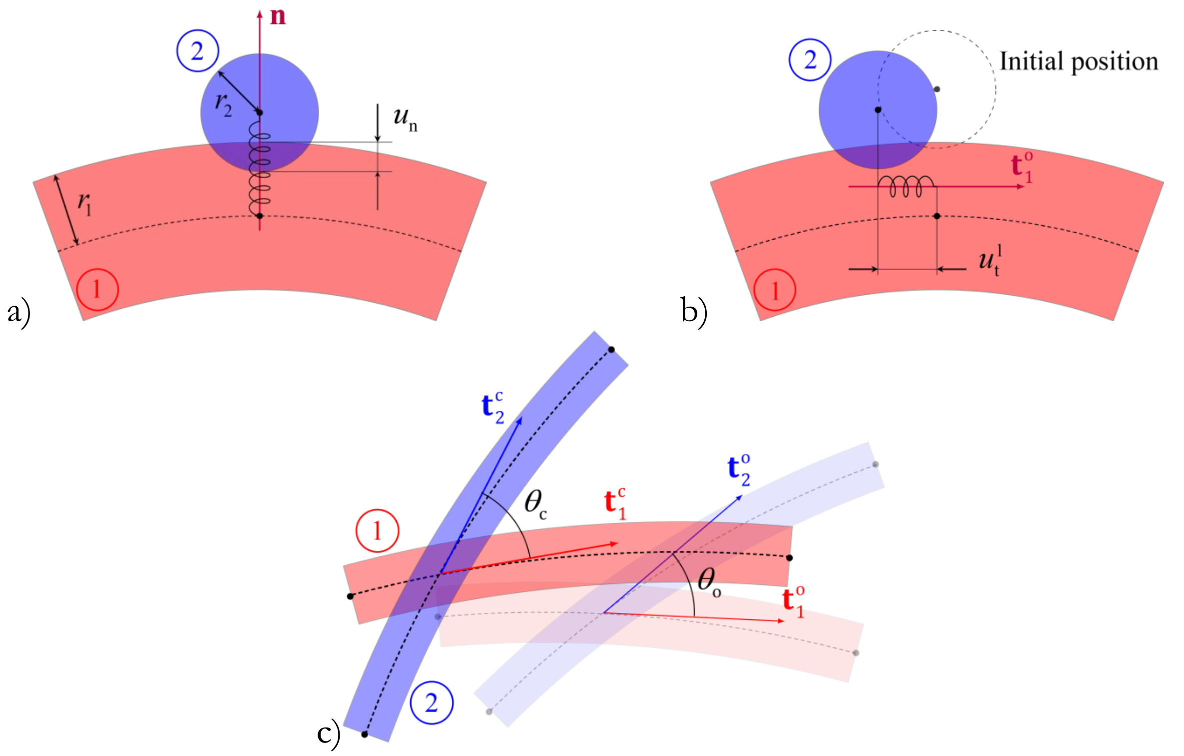

Fig. 5. Schematic representation of two crossing beams in contact and penalty stiffness directions in a) normal direction; b) tangent direction and c) rotational direction.

The second tangent slip u_t^2 is determined by projecting the computed slip along the slave beam to the directional vector $\mathbf{v}_1^o = \mathbf{t}_1^o \times \mathbf{n}$ orthogonal to the tangent to the master beam and to the normal contact. An equivalent tangential slip is computed as $u_t = \sqrt{(u_t^1)^2 + (u_t^2)^2}$ and is later used in the cohesive relations, described in Section 2.1.3. Similar to the in-plane rotational constraint, we constrain two other rotations at the point of contact. The contact rotations are not involved in the cohesive relations since the contribution from the contact moments to debonding is assumed to be significantly smaller than from contact forces as the fibers are immobilized by numerous constraints along their length.

The principle of stationarity of energy potential is used to derive the finite element equations for the contact. To do so, the variations of normal and tangential gaps need to be calculated and linearized. Using some simplifying assumptions described in (Motamedian, 2016a, 2016b), we used an inconsistent linearization method and derived the tangent stiffness matrix for the contact element. As we showed earlier, this inconsistent formulation resulted in a simpler implementation and proved to be more stable and converge in less number of iterations for our application, especially when the load step size increases.

It should be noted that due to the nature of the penalty method, a small interpenetration and elastic sliding will be present. As a result, the directions of the contact normal and tangents are updated at each iteration. During the deformation, the contact point can change location from one beam element to an adjacent one. In order to prevent convergence difficulties due to the changed normal upon such transition, we enriched the shape of contact surface using the adjacent nodes. This

was done in terms of 2 nodes located to the left and 2 nodes to the right from the given contact element, meaning that each contact element consisted of seven nodes in total. Similar methods are earlier employed by (Litewka, 2007). Alternative to this contact smoothing is using geometrically exact beams, which do not require such measures (Meier et al., 2016).

A certain number of contact pairs in the generated networks appeared to be close to being parallel. It is known that in such cases, the closest point projection method for finding the contact point may yield multiple solutions or not converge at all. In case of multiple solutions, the contact status of the affected beams can change several times, which causes formidable convergence problems. To address this, different strategies have been proposed in the literature (Chamekh et al., 2014; Duville, 2012; Meier et al., 2016), some of which include treating such contact points with a point-to-surface contact or having a distributed line force instead of a point force. In our case, we put an experimentally determined threshold of 2 degrees on the crossing angle between two beams and neglect all the contact pairs crossing at an angle below it. The estimated number of contacts removed due to this threshold was below 0.5% of the total number of contacts in the computed cases and was considered insignificant. If during the computation the closest point projection fails to yield a unique solution for a certain contact point, the fibers in that contact were considered debonded. The number of such cases summed up to less than 0.1% of all of the debonded contacts and had a negligible effect on the overall response of the network.

2.1.3. Bonds

We describe the mechanical bond behavior with traction-separation laws and model it with a cohesive zone model, which is based on the contact forces (the contact moments are not accounted for in the cohesive zone model). A mixed failure mode was . A schematic representation of the adopted bilinear cohesive zone model for the tangential direction is shown in Fig. 6a. In both the normal and tangential directions, denoted by subscripts n and t, respectively, the initial linear loading continues until the bond strength ($F_{\mathrm{t}}^{\max}$) is reached (at the normal separation and slip displacement $\overline{u}_{\mathrm{n}}$ and $\overline{u}_{\mathrm{t}}$, where the latter is the effective displacement for both tangential directions $\overline{u}_{\mathrm{t}} = \sqrt{u_{\mathrm{t1}}^2 + u_{\mathrm{t2}}^2}$); followed by a linear softening behavior until failure (with critical displacement $u_{\mathrm{n}}^{\mathrm{c}}$ and $u_{\mathrm{t}}^{\mathrm{c}}$). In this formulation, $u_{\mathrm{t}}^{\mathrm{c}} - \overline{u}_{\mathrm{t}}$ is the separation distance that reflects the bond damage in the tangential direction, which is 15% larger than that distance at strength (the same is valid for the normal direction). A contact is considered to be fractured when it reaches $F_{\mathrm{t}}^{\max}$ and separated/debonded when it attains the critical displacement.

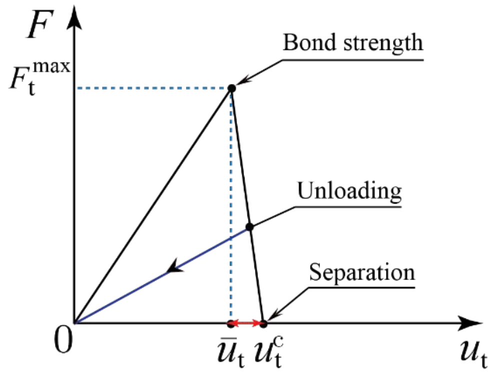

Fig. 6. The cohesive bond failure model in terms of a traction-separation diagram with linear softening, shown for the tangential direction. Unloading subsequent to damage follows the secant towards zero force.

A linear traction-separation law includes the normal (F_n) and the equivalent tangential (F_t) force components:

$$\begin{cases} F_n = K_n u_n (1-D) \\ F_t = K_t u_t (1-D) \end{cases}, \quad (3)$$

which are coupled through the damage parameter D. In order to account for the contribution from each direction into the separation through this damage parameter, we use a non-dimensional effective displacement β, which is defined through the separation- and slip-ratios as follows:

$$\beta = \sqrt{\left(u_n / \bar{u}_n \right)^2 + \left(u_t / \bar{u}_t \right)^2}, \quad (4)$$

where u_n and u_t are the calculated normal separation and tangent slip respectively (Fig. 6). The damage onset β always exceeds 1, which effectively corresponds to the second power law energy criterion, if this condition is rewritten in terms of critical fracture energies. At the onset of damage, the damage parameter D is equal to 0 and reaches 1 at the point of separation. In order to force the damage parameter D to be compatible with a single mode failure, the following linear relationship is adopted:

$$D = \frac{\beta - 1}{\beta} \left(\frac{u_n^c}{u_n^c - \bar{u}_n} \right). \quad (5)$$

Finally, in order to achieve separation in tangential and normal direction simultaneously, the normal contact stiffness was scaled as it is shown in Equation 5,

$$K_{\mathrm{n}} = K_{\mathrm{t}} \cdot F_{\mathrm{n}}^{\max} u_{\mathrm{t}}^{\mathrm{c}} \Big/ F_{\mathrm{t}}^{\max} u_{\mathrm{n}}^{\mathrm{c}} \tag{6}$$

to satisfy the following constraint:

$$\left(\frac{u_{\mathrm{n}}^{\mathrm{c}}}{u_{\mathrm{n}}^{\mathrm{c}} - \bar{u}_{\mathrm{n}}} \right) = \left(\frac{u_{\mathrm{t}}^{\mathrm{c}}}{u_{t}^{c} - \bar{u}_{\mathrm{t}}} \right). \tag{7}$$

Since we assume linear damage evolution, any unloading after the damage initiation is always linear towards zero force and the value of the damage parameter before unloading is preserved in the subsequent reloading.

The characteristics of bonds are summarized in Table 3. We assume a constant normal to the tangential ratio for bond strength at 0.25 in all the numerical tests. It should be noted that the contact parameters in the normal direction influence the results marginally, even when varied by a factor of 10 with respect to the corresponding parameters in the tangential direction. This is a manifestation of the fact that the overwhelming majority of the fibers fails in nearly pure shear mode in the considered cases.

Table 3. Characteristics of bonds used in the network simulation.

	Tangential direction	Normal direction
Bond strength, mN	11.00	2.75
Bond stiffness, 10^9 N/m	8.90	8.00
Separation distance, μm	1.56	0.35

2.2. Network generation

A three-dimensional network of fibers is numerically generated with deposition techniques (Kulachenko and Uesaka, 2012) with a custom code. To summarize the formulation, a number of specific features are considered:

1) *Fiber geometrical properties.* A fiber is represented as a series of beam elements.

The fiber geometry can either be selected randomly from the characterized pulp, or follow a prescribed length-weighted distribution. In either case, the data for fiber cross-sections acquired from FMA are corrected through μCT measurements; see Table 1.

2) *Fiber deposition.* In order to create a symmetrical density profile with respect to the mid-surface, fibers are deposited on an imaginary flat surface from both sides. This method of fiber deposition is different compared to the network generation procedure in our earlier works (Borodulina et al., 2012; Borodulina et al., 2015), in which the deposition took place from one side only. The reason for this change is the relatively symmetric density profile of the handsheets discovered in the microtomography analysis, which cannot be recreated by depositing fibers from one side only. The fiber orientations and positions are assumed to be completely random, in order to secure the isotropic in-plane orientation of the fibers. A generated fiber is allowed to fall down on already-deposited fibers on the respective

side of the mid-plane. The contacts with other fibers are through the contact search algorithm.

During the deposition, the fibers are allowed to bend and wrap around other underlying fibers. The cross-section of the beams was oriented to have the height aligned with thickness direction before being deposited. A parameter referred to as the interface angle φ (Fig. 7), was used to control the degree to which the fibers can bend during the deposition. The interface angle, together with the WH ratio, affects the thickness of the network. In addition, we introduced a compressibility parameter that can be used to adjust the thickness of the network to the target value. This parameter was used to scale the coordinates of the fiber segments in the thickness direction and accounts for the fact that fibers can be compressed more in the bond region, mainly because the exact cross-section of the fiber in this region cannot be easily determined. We used both the compressibility parameter and the interface angle to match the spatial characteristics of the structure, as will be explained in the verification procedure.

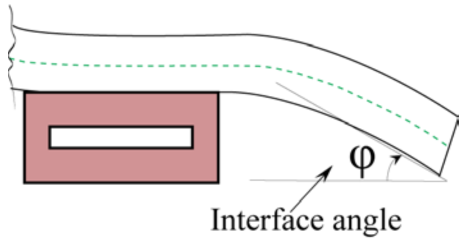

Fig. 7. Fiber interface angle as defined in the model.

*3) Network thickness***.** Since we work with a 3D network, the unevenness of the surfaces complicates the definition of the sheet thickness due to its roughness. The thickness is affected by fibers' WH ratio (Fig. 4g) and/or interface angle (Fig. 7), both of which define how well the fibers are pressed/conformed against each other.

Within the presented model, we estimate the network thickness as follows. First, we calculate the average distance of all fiber segments $(\bar{z})$ from the imaginary plane at zero distance $(z=0)$:

$$\bar{z} = \frac{\sum_{i=1}^{n_f}\sum_{j=1}^{n_s}|z_{ij}|}{n_f n_s}, \tag{8}$$

where z_{ij} is the z - coordinate of the center of the *j*-th segment of the *i*-th fiber. The total number of fibers is n_f, each having n_s segments. Similarly, we calculate z - weighted average distance all fiber segments $(\bar{\bar{z}})$ from the same imaginary plane at zero distance:

$$\bar{\bar{z}} = \frac{\sum_{i=1}^{n_f}\sum_{j=1}^{n_s}(z_{ij})^2}{\sum_{i=1}^{n_f}\sum_{j=1}^{n_s}|z_{ij}|}. \tag{9}$$

Hence, the thickness of the network is approximated as

$$\text{Network Thickness} \approx 2(\bar{z} + \bar{\bar{z}}). \tag{10}$$

Using this method, we estimate the mean thickness of the scanned networks to be 57 μm. Although this value does not match exactly that measured with a caliper tool on the experimental sheets (44.9 μm), the proposed measure offers a reproducible way of comparing the thicknesses of the fiber networks.

4) Network size. Both the fiber network strength and stiffness are size-dependent. While the size dependency in strength has a complex nature and follows a weakest-link scaling law starting from certain size (Hristopulos and Uesaka, 2004), the size dependency in stiffness depends mostly on the average length of the fibers and appropriate boundary conditions. The representative size of a network was chosen from the size dependency tests, based solely on the network stiffness, to be 10 mm × 4 mm. We confirmed experimentally that the relative difference between the stiffness of a 10 mm × 4 mm sample and that of a 20 mm × 20 mm specimen is less than 2%. A sample of the numerically generated network of fibers with isotropic orientation and basis weight of 27 g/m^2 is presented in Fig. 8. The fibers, both with solid and open cross-sections, are visible in the thickness profile (Fig. 8b). The fraction of fibers with collapsed cross-sections is approximately 50%.

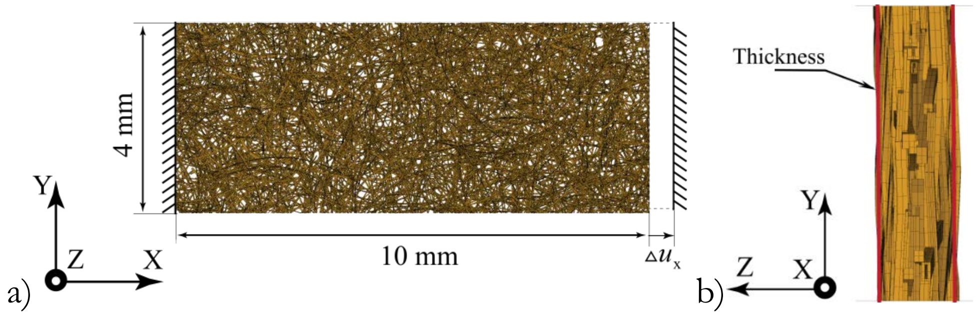

Fig. 8. Generated network (size 10 mm × 4 mm, basis weight of 27 g/m^2, thickness 57 μm): a) in-plane fiber orientation, b) through-thickness profile. Network thickness is defined in between the red lines according to the proposed thickness calculation method.

2.3. Evaluating the results of the simulation

We use the following nomenclature. Paper *strength* (MPa) is the maximum point on the stress-strain curve, shown in Fig. 9, with corresponding strain value called

paper *stretch* (%). The slope of the initial linear part of the stress-strain curve is as *stiffness* (GPa). The simulations are interrupted after at least 5% reduction in force from the peak region. This softening region is shown by a gray line in Fig. 9.

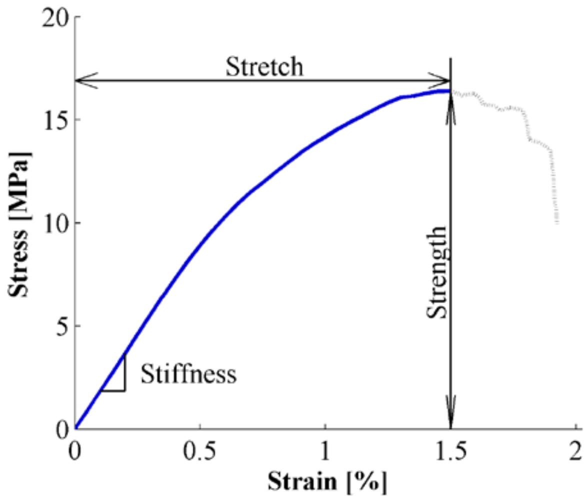

Fig. 9. Stress-strain curve of the reference network with constant bond strength. Definition of "Strength", "Stretch" and "Stiffness" nomenclature used throughout the article. The gray line represents the strain softening region.

3. Results and Discussion

3.1. Verification of numerical model

We verified the numerical model using experiments conducted earlier (Borodulina et al., 2012). The extracted cross-section properties and 3D structure of the sheet (Borodulina et al., 2016) allowed us to fine-tune the deposition model in order to match the connectivity data and density profile of the sheets. These sheets were first mechanically tested and later scanned with X-ray microtomography. The experimental data were available for these laboratory sheets made of well-characterized pulp. The sheets had the dimensions of 10 mm × 4 mm × 44.9 μm and grammage[††] of 27 g/m^2. A relatively small size of the sheets made it possible to perform direct simulation on the numerical samples having the same size.

We used three measures to ensure that the deposition model generated a representative network. By manually fitting to these measures using the parameters of the deposition model, i.e. the interface angle and compressibility coefficient, we ensured that the generated networks are fairly close to the sheets used in the experiments. These three measures are:

1) Network thickness and a number of contacts (NoC) per unit area. We can match them exactly. However, these are two scalar parameters, therefore, they do not give much information about 3D connectivity inside the network.

[††] Grammage is basis weight, defined as mass per unit area, usually expressed in units of grams per square meter.

2) Through-thickness density profiles (Fig. 10). A similar porosity profile was previously reported by Bloch et al. (2006) who performed simple optical profilometry of different paper grades. Porosity can be seen as inversely proportional to density.

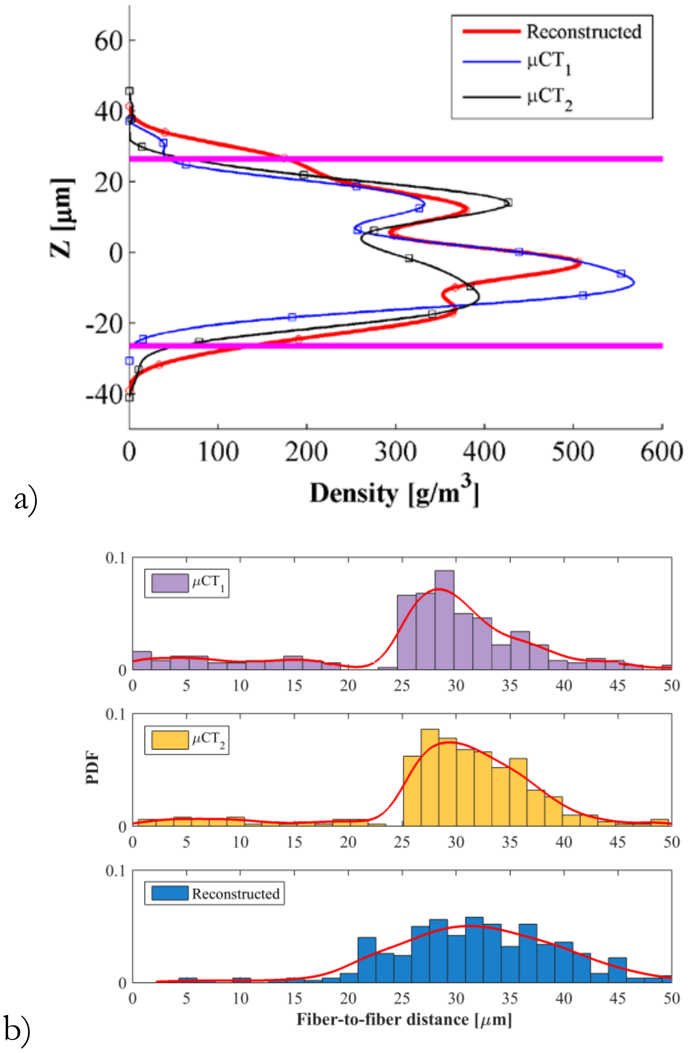

Fig. 10. Comparison of the reconstructed network properties with those extracted from two μCT samples denoted as μCT_1 and μCT_2 respectively. a) Density profile. b) Distance distribution between non-bonded fibers crossing in the top view.

3) Distances between unconnected fibers that cross in the top view. These distances are measured in the thickness direction and characterize a spatial arrangement of the fibers in 3D. The results on fiber-to-fiber distances from μCT samples and reconstructed networks are presented in Fig. 10b. The results from the generated network compare well to those extracted from μCT in terms of the mean value of the distance and the shape of the distribution. A relatively large number of fibers that have short distances in μCT measurements can be explained by missed contacts from the contact identification algorithm during processing of μCT images (Wernersson et al., 2014).

For the final verification with the experimental tensile test, we simulated the stress-strain curve of the reconstructed network. The conversion from a specific stress (kN·m·kg^{-1}) of the experimental data to traditional stress values in MPa was performed using the sample thickness measured with μCT (44.9 μm). The numerical experiments were displacement-controlled and the boundary conditions were similar to those used in the physical experiments performed on the cut samples of identical size. The nodes crossing the left boundary were constrained in all 6 degrees of freedom (3 translational displacements and 3 rotations). At the right boundary, all the degrees of freedom were constrained apart from the translational displacement u_x, which was gradually increased until the sufficient softening in the tensile response was reached (Fig. 8a). The considered network structures were not periodic and the free boundaries in the cross-direction remained unconstrained. The results of the comparison are presented in Fig. 11.

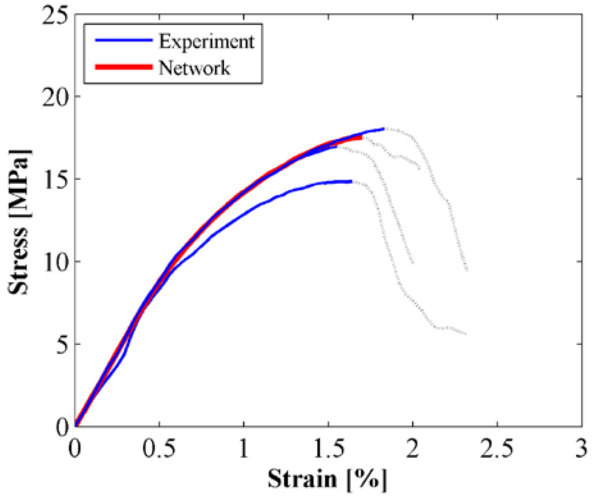

Fig. 11. Stress-strain curve of the experimental laboratory sheets (Borodulina et al., 2012), compared to the reference network with constant bond strength.

As can be seen in Fig. 11, all the parameters (i.e. tensile stiffness, strength, and stretch) are well captured with the chosen fiber and bond constitutive properties presented earlier in Table 2 and Table 3, as well as with the reconstructed geometry of the network.

Without changing the properties of the generation procedure, we also compute the network of greater grammage of 65 g/m^2 and compare to the experimental results obtained by the standard tensile test on 100 mm × 15 mm strips. The results of this comparison are summarized in Table 4.

Table 4. Comparison of experimental and network simulation data for sheets with a grammage of 65 g/m^2.

Stiffness, GPa		Strength, MPa	
experiment	simulation	experiment	simulation
3.21	2.82	28.1	31.54

The numerical results show good agreement with the experiment. Note, the physical size of the experimental sample was larger, which explains the lower strength of the sample.

3.2. Effect of variation in fiber properties

The pulp data are usually expressed in length-weighted averages. These values are used for assessing the quality of the pulp and for benchmarking purposes. In this section, we assess whether using the length-weighted values is a justified strategy in relation to their impact on the mean values of the strength and stiffness of the networks.

In our numerical tests, we worked with the sheets generated by the deposition technique. The reference networks are based on data from the original pulp, characterized with respect to their geometrical properties, as discussed in the previous section. We compared the mean values of tensile strength, stiffness, and a stretch of the networks made of the original pulp with the networks where fibers had equal properties, such as length, diameter, wall thickness and shape factor. In every case, we considered five network realizations having the same (length-weighted) mean fiber properties with isotropic orientation.

In the study of fiber length distribution, the size of the computed network affected the fiber length distribution through "cutting" the fibers at the boundaries. Therefore, achieving a sharp distribution with a limited size is infeasible. Fig. 12 shows the length distribution in the sheet made of the original pulp and that made of fibers having the same length. Both networks were numerically cut to the size of 10 mm × 4 mm from a larger sample. It is possible to observe the fraction of cut fibers in the corresponding histogram. However, it is clear that the distribution is more uniform with the largest fraction of fiber volume having the same length.

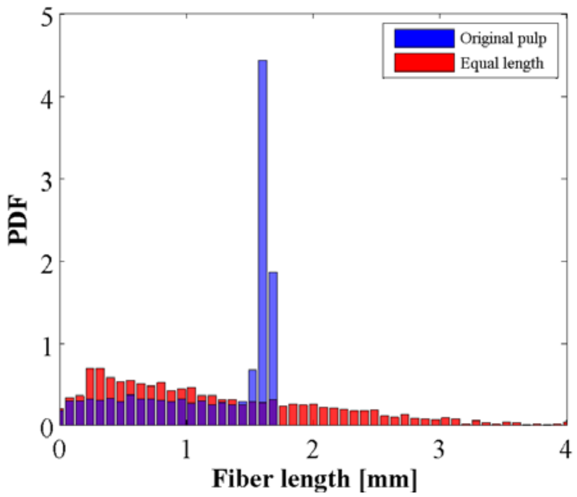

Fig. 12. Fiber length distribution in the cut sample for the study case "Equal fiber length," compared to the original fiber length distribution in the pulp.

In Table 5, we present the values of fiber properties that were kept constant during individual study cases. These were taken from the data of the considered pulps and specified earlier in Table 1.

Table 5. Specified variation in fiber properties used in the simulations. In all study cases, only one parameter was kept constant, the value of which is indicated in the corresponding column. The other parameters were subject to their natural variation according to the measured data.

Equal length, mm	Diameter, μm	Wall thickness, μm	Shape factor, [-]
2.34	23.83	3.96	0.945

The results of the simulated stress-strain curves of different fiber properties are shown in Fig. 13 for two different grammages, 27 and 65 g/m². Each curve represents the mean of five simulated networks with the standard deviation shown by error bars: vertical for stress and horizontal (in bold) for strains. The softening parts of the curves were discarded. For an easier understanding of the trends, we have illustrated the results with the help of the bar plots in Fig. 13, showing the relative difference in results with respect to (w.r.t.) the reference case based on the original, unmodified pulp.

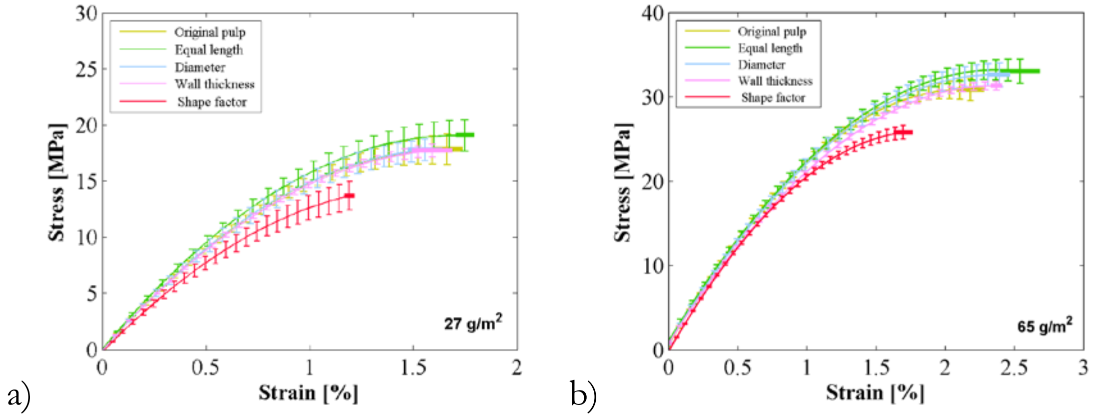

Fig. 13. Stress-strain curves for networks with different fiber properties according to Table 6, shown for a) 27 g/m² and b) 65 g/m². Vertical error bars characterize standard deviation in stress, while the standard deviation in strain is presented by a thick horizontal error bar.

Fig. 13 and 14 indicate that the fiber shape factor and fiber length equalization have the largest impact on both the strength and stiffness of the network, while other fiber parameters do not affect the average network properties to the same extent. Similar trends for length and shape factors are seen for both lower and higher grammages. Removing cross-sectional variation gave a positive impact only for the higher grammage. From Fig. 14a, it can be seen that fixing certain fiber geometrical properties during network generation changes the number of contacts. To single out the effect of the change in the number of contacts for the other three cases (i.e. “Diameter”, “Wall thickness” and “Shape factor”), we assigned the same number of contacts in all the considered networks. After computing the initial position of the contacts prior to applying the load, a small fraction of the excessive contact points was removed uniformly across the networks to arrive at exactly the same number of contacts in all the generated networks. The removal was

performed by removing the corresponding contact pairs and all the contact elements associated with them prior to computations. This ensured that the fibers from the removed bonds could no longer be in contact during the analysis. Fig. 14b shows the results in the case when the number of contacts was the same on all the networks. Fixing the number of contacts in the network decreases the effect of the length and shape factor, proving that the observed differences both in strength and stiffness were caused by the changed number of contacts.

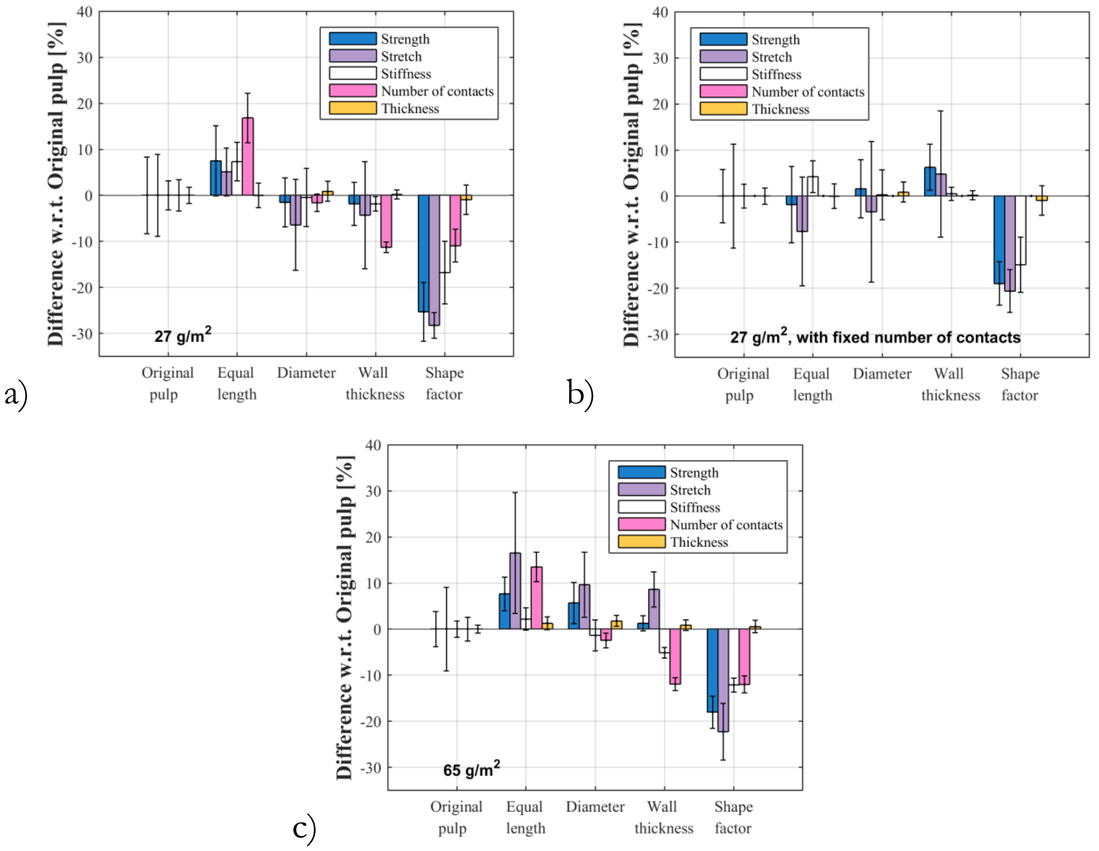

Fig. 14. Effect of the distribution of fiber properties on the average network properties (i.e. strength, stiffness, strain at failure (stretch), network thickness and number of contacts) with a) & c) original and b) fixed predefined number of contacts, shown for a) & c) 27 g/m² and b) 65 g/m². The relative difference in the average properties is calculated with respect to the "Original pulp" values used as a reference.

To a large extent, the effect of the shape factor (manifested in the reduction of network stiffness, and strength) remained unchanged, even after we eliminated the effect of the number of contacts. This can be explained by the fact that in networks composed of fibers that have the same shape factor, a greater fraction of energies is stored in bending type of deformation and in contact regions. Fig. 15 shows how the percentage of longitudinal, bending and contact energy changes during the deformation in "Original" and "Shape factor" test cases. (A fraction of shear energy stored in fibers was negligible.) Compared to the original case, the percentage of energy stored in longitudinal deformation of the fibers was decreased by 20% and shared between the bending deformation and contact regions, in particular, prior to failure. As bending is a more compliant form of deformation, it is reflected in the decreased stiffness. At the same time, more energy is stored in

contacts, meaning the contacts experience greater stress levels; which is the cause of lower network strength.

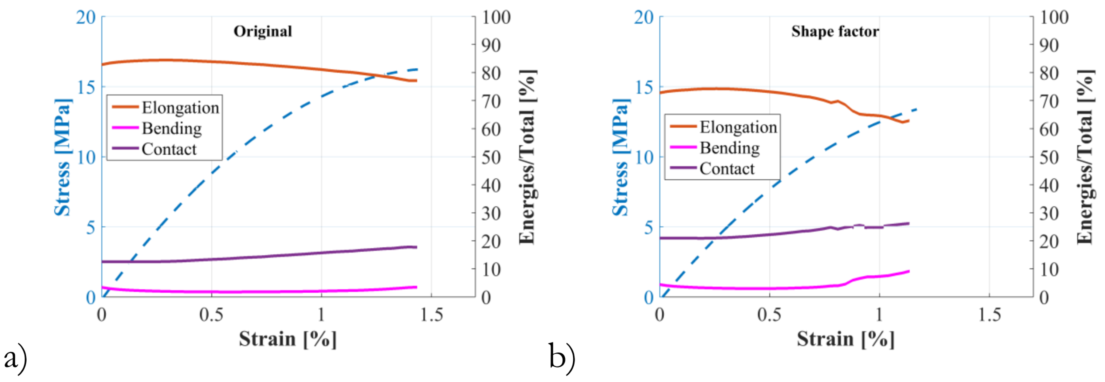

Fig. 15. The fraction of energies with respect to the total energy along the averaged stress-strain curve, shown for a) "Original" and b) "Shape factor" test cases for networks of 27 g/m² with fixed number of contacts.

The coefficient of variation (COV) for the considered cases is presented in Table 6.

Table 6. Effect of variation of fiber parameters on the properties of networks of 27 g/m² and 65 g/m² grammages. The lowest and highest values are emboldened and colored differently.

	COV in Strength [%]	COV in Stretch [%]	COV in Stiffness [%]	COV in NoC [%]	COV in Thickness [%]
**27 g/m²**	a) With number of contacts by contact search algorithm				
Original pulp	**8.31**	8.92	3.13	3.43	1.73
Equal length	7.63	5.19	4.18	**5.36**	2.69
Diameter	5.32	9.84	6.30	1.87	2.15
Wall thickness	**4.67**	**11.63**	**1.54**	**1.15**	**0.96**
Shape factor	6.41	**2.80**	**6.80**	3.56	**3.19**
**27 g/m²**	b) With fixed number of contacts equal to the Original pulp case				
Original pulp	5.76	11.30	2.57	0	1.73
Equal length	**8.28**	11.82	3.42	0	2.69
Diameter	6.30	**15.24**	5.40	0	2.15
Wall thickness	5.00	13.69	**1.44**	0	**0.96**
Shape factor	**4.74**	**4.63**	**5.98**	0	**3.18**
**65 g/m²**	c) With number of contacts by contact search algorithm				
Original pulp	3.78	9.03	1.74	2.56	**0.85**
Equal length	3.66	**13.10**	2.39	**3.20**	**1.38**
Diameter	**4.45**	7.02	**3.38**	1.61	1.19
Wall thickness	**1.62**	**3.80**	**1.15**	**1.39**	1.14
Shape factor	3.48	6.14	1.53	1.81	1.33

Interestingly, the original pulp gave the highest variation in strength for the low-grammage network with the original number of contacts. At the same time, assigning a uniform fiber wall thickness gave the lowest variation in strength for both the low and high grammage networks. The coefficient of variation was reduced by the increased grammage for all the properties, which emphasizes the role of the thickness for strength variations. It should be noted that the number of

considered samples was insufficient to draw solid conclusions concerning the effects on strength variations; this question will be addressed separately.

3.3. Effect of the variation in bond strength

Fibers are bonded in the network in a very complex process: the bond strength depends on a number of factors, including the fiber type, fiber surface roughness, fiber alignments and eventual details of the bond geometries. A large variation in data for the measured bond strength in artificially created bonds has been reported. The values for bond strength vary in the range of 1–60 mN (Magnusson et al., 2013; Fischer et al., 2013). Remarkably, a large number of artificially created bonds showed a rather low value of the bond strength; the results of bond strength measurements resembled a gamma distribution. The experimental findings presented in the literature raise the question as to the importance of the distribution of bond strength compared to mean values.

To address this question, we performed a controlled numerical study in which we tested five cases of various bond strength distributions:

1) The reference case with a uniform bond strength of 11 mN across all the bonds in the network.
2) The network with the bonds having a strength that varied according to a Gamma distribution, yet having a mean value of 11 mN and standard deviation of 5.45 mN.
3) Same as 2) but with a truncated Gaussian distribution, using the same mean value and standard deviation of 11 mN and 5.45 mN, respectively.
4) An L-shaped gamma distribution with a mean value of 11 mN and standard deviation of 10 mN.
5) Random distribution with the same mean value of bond strength of 11 mN.

In all the tests, we kept the network structures the same; that is, we considered the effect of the changed bond properties with a set of five networks per each distribution.

The considered distributions from cases 2–5 are shown in Fig. 16. Note that cases 3 and 4 resemble the measured bond strength distribution for bonds with and without strength additives presented by Marais et al. (2014).

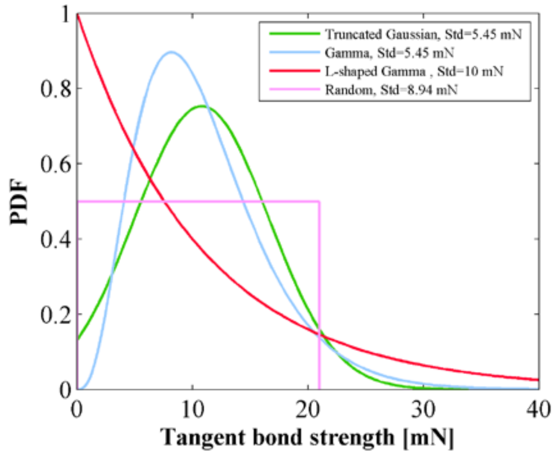

Fig. 16. Distributions for tangential bond strength utilized in the simulation based on experimentally measured bond strength values of Marais et al. (2014). A constant normal to the tangential ratio for bond strength is assumed to be 0.25 in all the numerical tests.

Fig. 17 shows the stress-strain response of networks with the considered four different bond strength distributions compared to the reference case having constant bond strength, computed for low and high grammage sheets. Each curve represents the mean of five simulated networks with the standard deviation shown by error bars. The interval for recorded stretch is shown with a bold horizontal line. Remarkably, despite the difference in bond strength distributions and standard deviations, the computed mean strength was nearly the same, considering that the mean value of the bond strength is retained. The only exception is the L-shaped Gamma distribution of bond strength, which yielded a noticeably lower strength of the networks. Similar results were observed for handsheets of never-dried, unbleached kraft fibers from spruce, with bond strength following a comparable distribution shape with an observable difference in strength of up to nearly 50% compared to a different bond strength distribution (Marais and Wågberg, 2012). The trends seen in the lower grammage sheets are preserved in the higher grammage sheets. These results are presented in Fig. 18 in the form of relative difference (in percentages) for strength, stiffness and stretch with respect to the case of uniformly constant bond strength.

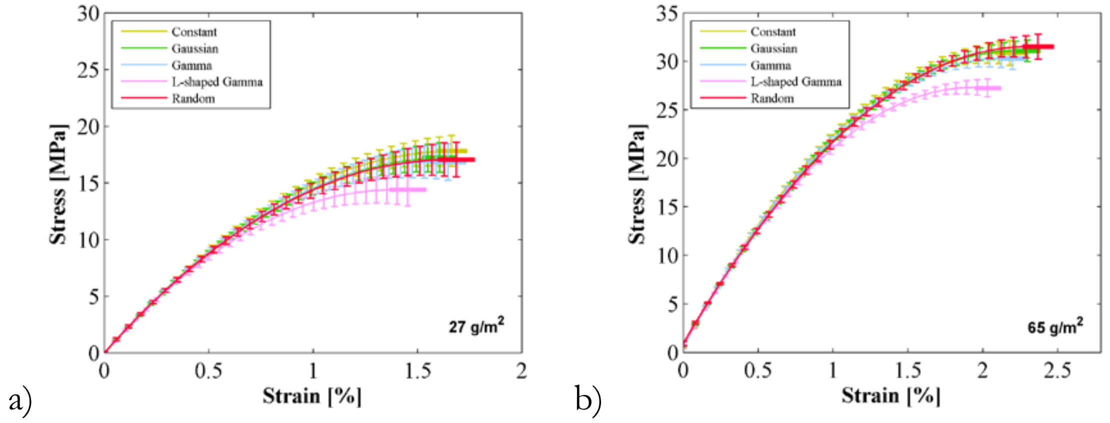

Fig. 17. Stress-strain curves for the networks with different distributions of bond strength according to Fig. 16, shown for a) 27 g/m^2 and b) 65 g/m^2. Vertical error bars characterize stress, while the standard deviation in strain is presented by a horizontal error bar in bold.

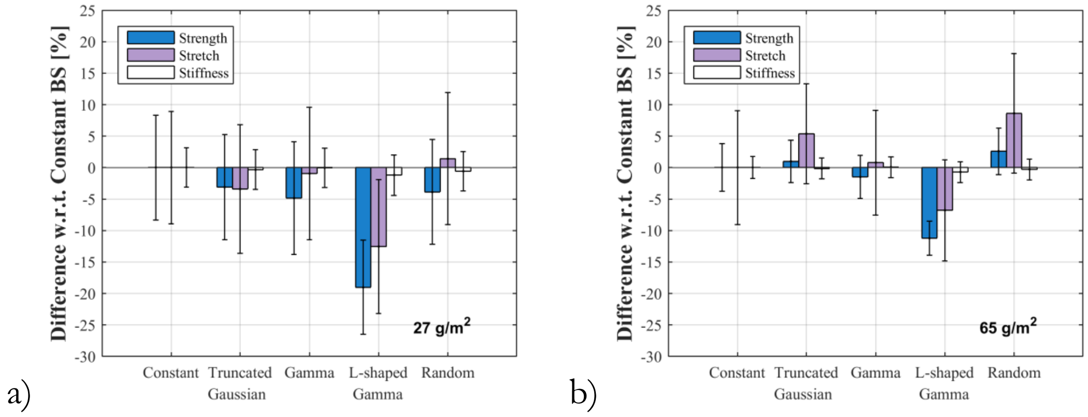

Fig. 18. Effect of bond strength distribution on the average network properties, shown for a) 27 g/m² and b) 65 g/m². The relative difference in the average properties is calculated with respect to the "Constant bond strength" values.

It can be seen from Fig. 18 that the stiffness of the network is completely unaffected. Only the L-shaped Gamma distribution led to a considerable reduction in strength (15–20%) for networks of the considered grammages, while the influence of the other distributions was not significant. It should be noted that the number of contacts remained unchanged in all these cases. The L-shaped Gamma distribution resembles that recorded by Marais et al. (2014). However, judging by the degree by which it changed in the simulations, we can conclude that the nearly 50% difference in strength observed on the sheet level by Marais and Wågberg (2012) cannot be explained by differences in the measured distributions alone.

In Table 7, the computed coefficients of variations in the bond strength variations case studies are presented. Interestingly, the coefficient of variations decreased in strength and stiffness but remained almost the same in the stretch with increased grammage.

Table 7. Effect of the variation of fiber-to-fiber bond parameters on the properties of networks of 27 g/m² and 65 g/m² grammages. The lowest and highest results are emboldened and colored differently.

	COV in Strength [%]	COV in Stretch [%]	COV in Stiffness [%]
**27 g/m²**			
Constant bond strength	8,30	**8.92**	3.13
Truncated Gaussian	8.35	10.21	3.15
Gamma	**8.97**	10.53	3.13
L-shaped Gamma	**7.46**	**10.63**	**3.20**
Random	8.32	10.49	**3.11**
**65 g/m²**			
Constant bond strength	**3.78**	9.03	**1.74**
Truncated Gaussian	3.37	**7.92**	1.66
Gamma	3.44	8.33	1.65
L-shaped Gamma	**2.71**	8.01	1.65
Random	3.70	**9.50**	**1.64**

In order to assist further interpretation of the difference in results between distributions, we first consider the rate at which the bonds broke under the selected

distributions of bond strength. The number of broken bonds can be related to the intensity of network fracture. Experimentally, this parameter can be approached, for example, by acoustic emission methods applied during paper straining, where the bond breakage is recorded by energy release (Salminen et al., 2002; Henriksson et al., 2008; Gradin et al., 2008). This method records very few events prior to the final fracture. However, the interpretation of experimental results is not straightforward, since the method is often unable to pick up events with low emitted energies. In our numerical methods, we can observe the number of fractured bonds even at the very beginning of network fracture.

We estimate the number of debonded contacts with respect to the total number of contacts (kept constant for all the networks) as a function of the applied tensile strain, presented in Fig. 19.

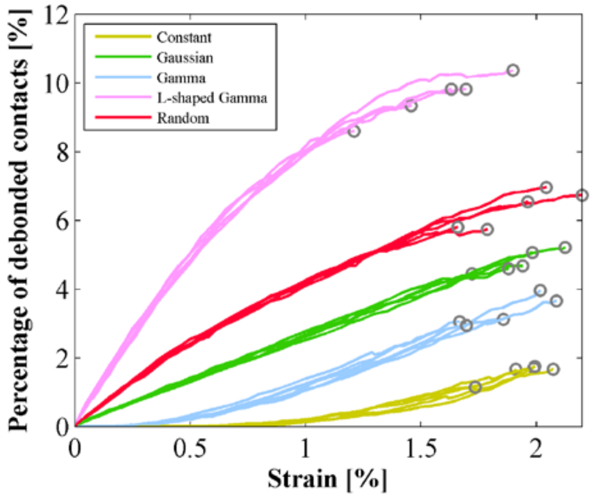

Fig. 19. Percentage of completely debonded contacts; circle represents the point of network failure.

Despite the fact that all the considered distributions resulted in more or less the same strength, the processes on the bond level, controlling the damage, were very different. In the network with a Gaussian distribution of bond strength, the bonds started to fail immediately with the applied strain at an almost linear growth rate. As expected, the largest number of debonded contacts occurs in networks with an L-shaped Gamma bond strength distribution with a large portion of weak bonds. In contrast, the network with a uniform constant strength had a relatively small fraction of bonds broken prior to failure, and the growth rate was exponential.

Another question that requires separate discussion are the observed differences in the mean stretch, which is an important factor in many applications. We compared the strain fields in the networks. This was effected by extracting the nodal displacements for fiber segments and mapping them on a regular square mesh, acting as a coordinate grid. The mapped displacement field is then smoothed over all the data points using a Butterworth filter. We compared the same networks computed with constant bond strength and L-shaped Gamma bond strength distributions, at a point of maximum stress on the stress-strain curve: Fig. 20c.

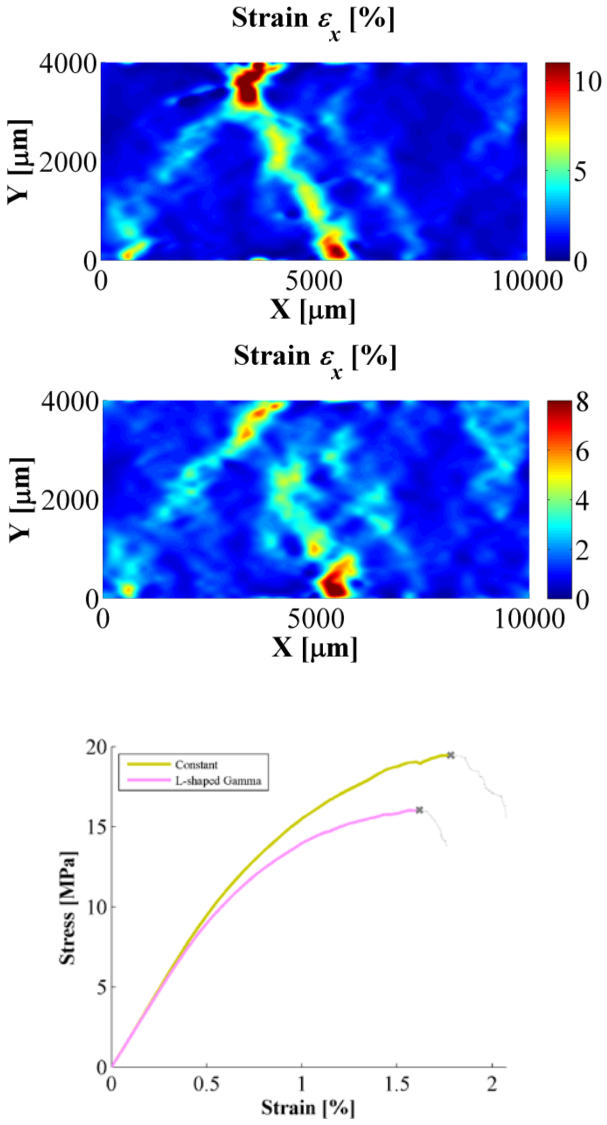

a)

b)

c)

Fig. 20. Strain field in the fiber network of 27 g/m², shown for two cases: a) constant bond strength, and b) varied bond strength according to the L-shaped Gamma distribution. The color bar represents the strain values. The results were collected upon reaching the network strength at the point marked by "x" in c).

Since the difference in the two numerical study cases, presented in Fig. 20a and 20b, is only in the bonding properties (constant vs. varied bond strength, keeping the same mean values), we can conclude that the fracture path is not affected by the change in bond strength distribution. At the same time, the unsymmetrical distribution in fiber bond properties makes the intensity of the strain localization smaller at the point of maximum stress along the stress-strain curve. This observation can be explained by the fact that the presence of a large number of weak bonds and their early failure allows for a more even strain distribution across the network.

4. Conclusions

We have approached the question of the effect of variability in the fiber and bond properties on the mean values of stiffness and strength of the network of fibers by conducting consistent numerical experiments using 3D models.

Our numerical model was verified against the experimental data, yielding a very good agreement. During the verification procedure, the numerical pulp (i.e. the assembly of fibers) was used to reconstruct the laboratory sheets. The cross-sectional geometry of the dry fibers was corrected using microtomography scans.

We found that the details of the strength distribution among the bonds in the network did not influence significantly the mean values of strength or stiffness of the network of fibers, as long as the distribution of bonds strength did not become extremely asymmetric with respect to mean value, such as in the case of a Gamma L-shaped distribution. This means that comparing the average bond strength having a sufficient number of samples is an adequate strategy, only if the bond strength distribution is close to being symmetric. The mean stretch is increased in the presence of bond variability, which is attributed to more uniform strain transfer during strain localization in the network when weak bonds fail prior to reaching the strength limit.

The length-weighted fiber properties are often used as quality criteria for paper pulp. We found that among the fiber geometries, the variability in length-weighted fiber shape factor and fiber lengths have a major effect. In particular, the network composed of fibers having the average length-weighted fiber shape factor constant had over 20% reduced strength and stiffness. This was partly due to the altered number of contacts, but mostly due to the increased contribution of bending deformation mode in the fibers. At the same time, reducing the length-weighted variation of fiber length had an observable positive effect on the average strength and stiffness. In the case of the fiber length, the effect is mostly attributed to the increased number of contacts as we found through equalizing their number across all the networks. Eliminating the variability in fiber cross-section brought little or no effect compared to the fiber length and the shape factor, even with the observed reduction in the number of contacts upon using the uniform distribution of fiber cross-sectional properties. The effect of the shape factor was found to be due to the increased percentage of energy stored in bending of fibers and in contact regions. This means that in benchmarking the characterization data, the variation in length and fiber shape factors should be given separate attention.

These conclusions are drawn for isotropic sheets with two different grammages, 27 and 65 g/m^2, composed of unrefined fibers.

Acknowledgments

We gratefully acknowledge the competence center BiMaC Innovation and WoodWisdom ERA-NET program (PowerBonds project) for financial support of this work.

References

Alava, M., Niskanen, K., 2006. The physics of paper. Reports Prog. Phys. 69, 669–723.

Bloch, J.-F., Rolland Du Roscoat, S., Mercier, C., Vernhes, P., Pineaux, B., Blayo, A., Mangin, P., 2006. Influence of paper structure on printability: characterisation of paper using X-ray synchrotron microtomography, in: 22nd International Conference on Digital Printing Technologies (NIP 22). Denver, United States.

Borodulina, S., Kulachenko, A., Galland, S., Nygårds, M., 2012. Stress-strain curve of paper revisited. Nord. Pulp Pap. Res. J. 27, 318–328.

Borodulina, S., Kulachenko, A., Tjahjanto, D.D., 2015. Constitutive modeling of a paper fiber in cyclic loading application. Comput. Mater. Sci. 110, 227–240.

Borodulina, S., Wernersson, E.L.G., Kulachenko, A., Hendriks Luengo, C.L., 2016. Extracting fiber and network connectivity data using microtomography images of paper. Nord. Pulp Pap. Res. J. 31.

Bronkhorst, C.A., Bennet, K.A., 2002. Deformation and failure behaviour of paper, in: Mark, R.E., Habeger, C.C.J., Borch, J., Lyne, M.B. (Eds.), Handbook of Physical Testing of Paper. Marcel Dekker, Inc., New York, pp. 313–427.

Carvalho, G.M., Ferreira, P.J., Martins, A.A., Figueiredo, M.M., 1997. A comparative study of two automated techniques for measuring fiber length. Tappi J. 80, 137–142.

Chamekh, M., Mani-Aouadi, S., Moakher, M., 2014. Stability of elastic rods with self-contact. Comput. Methods Appl. Mech. Eng. 279, 227–246.

Duville, D., 2012. Contact-friction modeling within elastic beam assemblies: An application to knot tightening. Comput. Mech. 49, 687–707.

Eriksson, M., Torgnysdotter, A., Wågberg, L., 2006. Surface modification of wood fibers using the polyelectrolyte multilayer technique: Effects on fiber joint and paper strength properties. Ind. Eng. Chem. Res. 45, 5279–5286. doi:10.1021/ie060226w

Fischer, W.J., Zankel, A., Ganser, C., Schmied, F.J., Schroettner, H., Hirn, U., Teichert, C., Bauer, W., Schennach, R., 2013. Imaging of the formerly bonded area of individual fibre to fibre joints with SEM and AFM. Cellulose 21, 251–260. doi:10.1007/s10570-013-0107-0

Gradin, P., Graham, D., Nygård, P., Vallen, H., 2008. The use of acoustic emission monitoring to rank paper materials with respect to their fracture toughness. Exp. Mech. 48, 133–137. doi:10.1007/s11340-007-9055-7

Hägglund, R., Isaksson, P., 2008. On the coupling between macroscopic material degradation and interfiber bond fracture in an idealized fiber network. Int. J.

Solids Struct. 45, 868–878.

Henriksson, M., Berglund, L.A., Isaksson, P., Lindström, T., Nishino, T., 2008. Cellulose nanopaper structures of high toughness. Biomacromolecules 9, 1579–1585. doi:10.1021/bm800038n

Heyden, S., 2000. Network modelling for the evaluation of mechanical properties of cellulose fluff. Ph.D. Thesis, Lund University.

Hirn, W., Bauer, U., 2006. A review of image analysis based methods to evaluate fiber properties. Lenzinger Berichte 86, 96–105.

Hristopulos, D.T., Uesaka, T., 2004. Structural disorder effects on the tensile strength distribution of heterogeneous brittle materials with emphasis on fiber networks. Phys. Rev. B 70, 64108-64108–18.

Ibrahimbegovic, A., 1995. On finite element implementation of geometrically nonlinear Reissner's beam theory: three-dimensional curved beam elements. Comput. Methods Appl. Mech. Eng. 122, 11–26.

Kulachenko, A., Uesaka, T., 2012. Direct simulations of fiber network deformation and failure. Mech. Mater. 51, 1–14. doi:10.1016/j.mechmat.2012.03.010

Litewka, P., 2007. Hermite polynomial smoothing in beam-to-beam frictional contact. Comput. Mech. 40, 815–826.

Litewka, P., Wriggers, P., 2002. Contact between 3D beams with rectangular cross-sections. Int. J. Numer. Methods Eng. 53, 2019–2041.

Magnusson, M.S., Zhang, X., Östlund, S., 2013. Experimental evaluation of the interfibre joint strength of papermaking fibres in terms of manufacturing parameters and in two different loading directions. Exp. Mech. 53, 1621–1634. doi:10.1007/s11340-013-9757-y

Marais, A., Magnusson, M., Joffre, T., Wernersson, E.G., Wågberg, L., 2014. New insights into the mechanisms behind the strengthening of lignocellulosic fibrous networks with polyamines. Cellulose 21, 3941–3950. doi:10.1007/s10570-014-0421-1

Marais, A., Wågberg, L., 2012. The use of polymeric amines to enhance the mechanical properties of lignocellulosic fibrous networks. Cellulose 19, 1437–1447. doi:10.1007/s10570-012-9712-6

Marulier, C., Dumont, P.J.J., Orgéas, L., Rolland du Roscoat, S., Caillerie, D., 2015. 3D analysis of paper microstructures at the scale of fibres and bonds. Cellulose 22, 1517–1539. doi:10.1007/s10570-015-0610-6

Meier, C., Popp, A., Wall, W.A., 2016. A finite element approach for the line-to-line contact interaction of thin beams with arbitrary orientation. Comput. Methods Appl. Mech. Eng. 308, 377–413.

Motamedian, H.R., 2016a. Robust formulations for beam-to-beam contact.

Licentiate Thesis.

Motamedian, H.R., 2016b. Beam2Beam, Matlab Central.

Nilsen, N., Zabihian, M., 1998. KCL-PAKKA: a tool for simulating paper properties. Tappi J. 81(5), 163–166.

Niskanen, K.J., Alava, M.J., Seppälä, E.T., Åström, J., 1999. Fracture energy in fibre and bond failure. J. Pulp Pap. Sci. 25, 167–169.

Paavilainen, L., 1990. Importance of particle size - fibre length and fines - for the characterization of softwood kraft pulp. Pap. ja puu 72, 516–526.

Page, D.H., 1969. A Theory for the Tensile Strength of Paper. Tappi 52, 674–681.

Räisänen, V.I., Alava, M.J., Nieminen, R.M., Niskanen, K.J., 1996. Elastic-plastic behaviour in fibre networks. Nord. Pulp Pap. Res. J. 11, 243–248.

Rohm, S., Hirn, U., Ganser, C., Teichert, C., Schennach, R., 2014. Thin cellulose films as a model system for paper fibre bonds. Cellulose 21, 237–249. doi:10.1007/s10570-013-0098-x

Salminen, L., Tolvanen, A., Alava, M., 2002. Acoustic emission from paper fracture. Phys. Rev. Lett. 89, 185503-185503–4. doi:10.1103/PhysRevLett.89.185503

Skowronski, J., 1991. Fibre-to-fibre bonds in paper. Part II: measurement of the breaking energy of fibre-to-fibre bonds. J. Pulp Pap. Sci. 17, 217–222.

Wang, H., Shaler, S.M., 1998. Computer-simulated three-dimensional microstructure of wood fibre composite materials. J. Pulp Pap. Sci. 24, 314–319.

Wernersson, E.L.G., Borodulina, S., Kulachenko, A., Borgerfors, G., 2014. Characterisations of the fibre networks in paper using micro computed tomography images. Nord. Pulp Pap. Res. J. 29, 468–475.

Wilbrink, D.V., Beex, L.A.A., Peerlings, R.H.J., 2013. A discrete network model for bond failure and frictional sliding in fibrous materials. Int. J. Solids Struct. 50, 1354–1363.
