# Supplementary material for: Stochastic Constitutive Model of Isotropic Thin Fiber Networks Based on Stochastic Volume Elements
Source: Materials (Basel). 2019 Feb 11;12(3):538. doi: 10.3390/ma12030538 (PMC6384688; doi:10.3390/ma12030538)
Supplement: Supplementary file 1 [file materials-12-00538-s001.zip › Suppli/ReadMe.docx]

Dear User,

A 3D network of fibers is generated using a deposition technique in which the fibers are sequentially deposited on a flat surface from two sides. The deposition algorithm can be outlined as follows:

1. The fiber geometry is chosen from the fiber characterization data acquired with FiberLab, which is an apparatus for measuring fiber characteristics. It contains length, width, height, wall thickness, and curvature. The curvature is represented through an arc of constant curvature located in a single plane parallel to the deposition plane. The cross-sectional data is corrected using microtomography scans of a paper produced using the considered softwood kraft pulp. The details of the correction are described elsewhere [1].
2. The fiber orientation is chosen either randomly in this work although it can be controlled to match a specific distribution.
3. The fiber position before deposition onto the domain is chosen randomly.
4. The first fibers are deposited on the flat plane consequently from above or below. For the subsequent fibers, we first find the intersection between them and the previously deposited fibers in the plane (Figure 1a).
5. The found intersection points are lifted discretely to exclude penetration (Figure 1b). The contact search diameter depicted in the figure corresponds to the height of the fiber, which is smaller or equal to the width of the fiber normally.
6. The fiber geometry is smoothed to remove discontinuities caused by the previous step (Figure 1c). During the smoothing, we control the maximum angle which the fibers can form, and it was set to 5 degrees in this study.
7. When the grammage (the basis weight or the weight per unit area) of the network is reached the prescribed value, the deposition procedure is stopped. The grammage used in this work was 28 g/m^2^, which is relatively low but corresponds to the set of handsheets used to calibrate the measurements in [1].
8. The thickness of the network is evaluated measured using the procedure described in [1]. After the thickness is brought to the target value by uniform scaling of the coordinates in the thickness direction with respect to the center plane of the network. The target value in this study was 68 micrometers, which corresponds to the measured and used in the calibration [1]. The scaling may result in interpenetration, which are zeroed out during the subsequent computations.


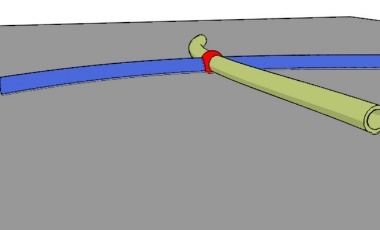


(

a

)


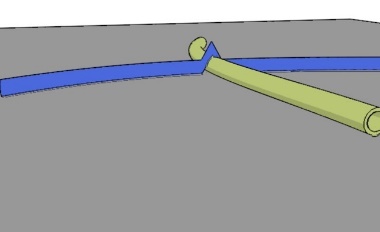


(

b

)


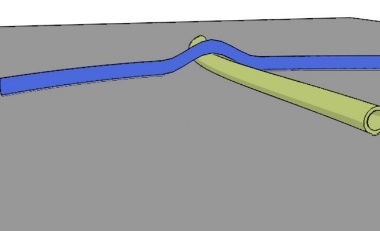


(

c

)

Figure 1: Steps in deposition procedure: (a) finding intersections with previously deposited fibers, (b) lifting the intersection point, and (c) smoothing the fiber.

After generation of the geometry, the fibers are represented with a fine mesh of curved segments and imported into a custom finite element code. They are converted into cubic splines and meshed with beam elements. Since the generation procedure contains the pulp-specific data, the software used for the fiber network reconstruction as well as the input data are uploaded as a supplementary material to the current paper.

[1] Borodulina, S., Motamedian H. R. and Kulachenko A. (2018) "Effect of fiber and bond strength variations on the tensile stiffness and strength of fiber networks." *International Journal of Solids and Structures*, 154(1), 19-32.

We have compiled the archive file which contains the stand-alone executable and examples. Let us give you a quick overview of what it contains:

1. MyPacking.exe – main executable file.
2. ModelingData.txt – input to the network generation module.
3. Stora32_clean.txt – results from PTS FiberLab analysis of StoraEnso32 pulp.
4. Results file from a test run.
   1. *.seed  – random seeds used in the analysis.
   2. *.dat – summary info on the generated network.
   3. *.mat – material properties (material number; elastic modulus; shear modulus). Note, they are fictitious here, we overwrote during the computations according to the table in the article.
   4. *.xyz – coordinates of the nodes (node number; x; y; z).
   5. *.nod – connectivity data (segment number; 1^st^ node; 2^nd^ node; 3^rd^ node; fiber number; material number).
   6. *.typ  – cross-section data (fiber number; type of the cross section (1-closed, 2 open);  width; height; wall thickness for the open fiber).
   7. *.dst  – distances between the fibers crossing as seen from the top (contact number; number of 1^st^ fiber in contact; number of 2^nd^ fiber in contact; natural coordinate of the contact points along the 1^st^ fiber; natural coordinate of the contact points along the 2^nd^ fiber; distance (zero means there are in contact)).
   8. *.con  – connectivity data (number of 1^st^ fiber in contact; segment number of 1^st^ fiber in contact; the number of 2^nd^ fiber in contact; segment number of 2^nd^ fiber in contact; x; y; z –coordinates of the contact).

Now about the input data file ModelingData.txt. There are many lines there and some of them are inactive if you use numerical furnish. It already has the inputs for the reference 24x24 mm case from the article. The results of the generation will appear in the same folder the program is executed from. The progress of the generation is reported on the screen.


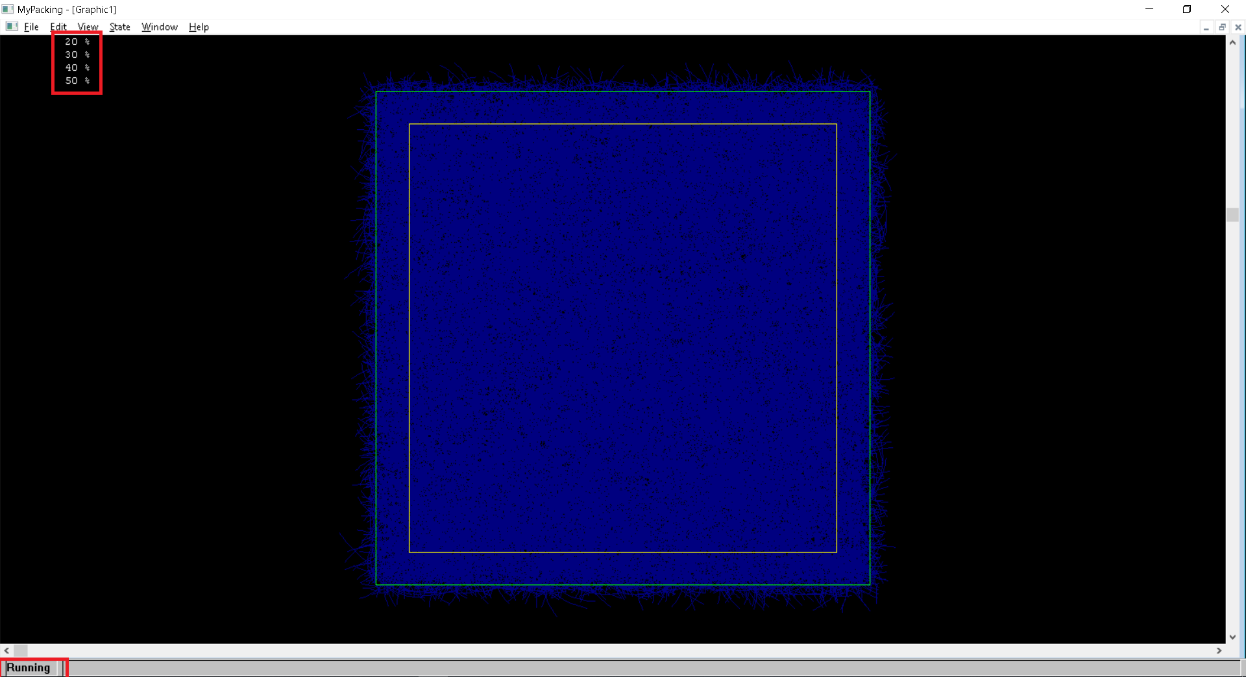


Figure 2: Screenshot from the network generation module.

The main control parameters during the generation are as follows:

**nSeries** – the number of random networks to generate

**UseMeasuredData**  – 0: Use random data;

1: Use data from measurement;

2: Use data from measurement with constant length equal to the length weighted average;

3: Use data from measurement with constant cross-section radius equal to the length weighted average;

4: Use data from measurement with constant wall-thickness equal to the length weighted average;

5: Use data from measurement with constant Curvature equal to the length weighted average;)

**MeasurementFile**  – filename for measured data

**RadSwell** – ratio between the dry and wet radius of fibers

**WThkSwell** – ratio between dry and wet wall thickness of fibers)

**MaxCurvature**  - maximum Curvature (Ratio between curved and projected length) to be read from FiberLab data. The fibers with larger curvature are ignored.

**MinFibLength**  - fibers with shorter lengths are treated as fines

**FinePercentage**  - between 0 to 100 for percentage based on the number; 0 to -100 for Percentage based on volume; bigger than 100: no constraint;

**Density** – fiber density

**WHRatio** – WH ratio (0 for a circular cross-section)

**MakeRectMethod** – 1: takes input width as diameter and matches the areas; 2: takes input width as width and calculates height using WHRatio

**WallThkPressability** – 0: Not pressable; 1:Completely pressable

**MinHollowRatio** – ratio between the minimum allowed hollow part dimension and wall thickness (holes with dimensions smaller than allowed will be removed and sections will become solid))

**InterfaceAngle**  - the maximum angle of a fiber-piece with horizontal plane

**AngleMean** – mean fiber orientation angle with respect to MD

**AngleStd** – standard deviation of the angle; 0: handsheet with completely random orientations

**ShapeMin** – 1: include sine-shaped fibers, 2:exclude sine-shaped fibers)

**ShapeMax** – 1: include polynomial-shaped fibers, 2:exclude polynomial-shaped fibers)

**Grammage** – Grammage

**NetworkLength** – Length of the sheet

**NetworkWidth** – width of the sheet

**PaperThk** – target paper thickness (if =0 it will not be pressed)

**Direction** – 1: one-sided network (deposition from top), -1: two-sided network(deposition from both sides))

**CloseAfterFinished** – close the batch window after generation .TRUE. or .FALSE.

**SaveContData** – produce the contact connectivity data .TRUE. or .FALSE.

Kind regards,

Artem

The support contact: artem@kth.se
